# Starvation Promotes Autophagy-Associated Maturation of the Testis in the Giant Freshwater Prawn, *Macrobrachium rosenbergii*

**DOI:** 10.3389/fphys.2019.01219

**Published:** 2019-09-27

**Authors:** Wilairat Kankuan, Chaitip Wanichanon, Federica Morani, Sirorat Thongrod, Rossella Titone, Tanapan Siangcham, Matilde Masini, Michela Novelli, Prasert Sobhon, Ciro Isidoro

**Affiliations:** ^1^Department of Anatomy, Faculty of Science, Mahidol University, Bangkok, Thailand; ^2^Laboratory of Molecular Pathology, Department of Health Sciences, Università del Piemonte Orientale “Amedeo Avogadro”, Novara, Italy; ^3^Department of Translational Research and New Technologies in Medicine and Surgery, University of Pisa, Pisa, Italy

**Keywords:** aquatic organism, reproduction, autophagy vacuoles, spermatogenesis, gonadal maturation

## Abstract

Autophagy is a degradative process of cellular components accomplished through an autophagosomal-lysosomal pathway. It is an evolutionary conserved mechanism present in all eukaryotic cells, and it plays a fundamental role in maintaining tissue homeostasis both in vertebrates and invertebrates. Autophagy accompanies tissue remodeling during organ differentiation. Several autophagy-related genes and proteins show significant upregulations following nutrient shortage (i.e., starvation). In our previous study, we found that in female giant freshwater prawns subjected to a short period of starvation autophagy was up-regulated in consonant with ovarian maturation and oocyte differentiation. Whether and how starvation-induced autophagy impacts on testicular maturation and spermatogenesis of the male prawns remained to be investigated. In this study, we analyzed the effects of starvation on histological and cellular changes in the testis of the giant freshwater prawn *Macrobrachium rosenbergii* that paralleled the induction of autophagy. Under short starvation condition, the male prawns showed increased gonado-somatic index, increased size, and late stage of maturation of seminiferous tubules, which contained increased number of spermatozoa. Concurrently, the number of autophagy vacuoles and autophagy flux, as monitored by transmission electron microscopy and the autophagic marker LC3, increased in the testicular cells, indicating that a short period of starvation could induce testicular maturation and spermatogenesis in male *M. rosenbergii* along with modulation of autophagy.

## Introduction

The giant freshwater prawn *Macrobrachium rosenbergii* is an economically important decapod extensively cultured in many countries of South-East Asia. In male *M. rosenbergii*, the reproductive organs consist of testes and vas deferens. There are several treatments to stimulate testicular maturation and spawning in male prawns. For instance, administration of 5-HT and of GnRH has been shown to promote spermatogenesis, as reflected by the increase of testis-somatic index, the diameter of seminiferous tubules and germ cell proliferation (Poljaroen et al., 2011; Siangcham et al., 2013). In addition, eyestalk – ablation in male could abrogate gonad inhibiting hormone (GIH), resulting in the upregulation of insulin-like androgenic gland hormone (Mr-IAG) in the androgenic gland, in the increases of testis size and in germ cell proliferation, thus implicating a relationship between the neurotransmitters, GIH, GnRH, and IAG in controlling spermatogenesis (Phoungpetchara et al., 2011). Short-term starvation can also result in gonadal maturation and increased gametogenesis, as shown in some invertebrates including *Drosophila melanogaster* and prawns (Barth et al., 2011; Kankuan et al., 2017). Starvation is an intense stimulation of the autophagic process, which was found to accompany gametogenesis in these invertebrates (Barth et al., 2011; Kankuan et al., 2017).

Autophagy is a lysosomal pathway for degradation of self-constituents playing a pivotal role in macromolecular turnover and tissue remodeling during embryonic development and organogenesis (Hale et al., 2013; Wada et al., 2014). Autophagy is classified into three types: macroautophagy, microautophagy, and chaperone-mediated autophagy (Glick et al., 2010). Macroautophagy (herein referred to as autophagy) is the prominent pathway induced under nutrient shortage in order to recover vital substrates from the degradation of redundant cellular components (Kaur and Debnath, 2015). The autophagy process starts with the formation of a double-membrane phagophore that surrounds target proteins or organelles that eventually seals and enclosing them in an autophagosome. Subsequently, the autophagosome fuses with lysosomes to form an autolysosome wherein the lysosomal hydrolytic enzymes degrade the sequestered autophagic cargo. Autophagy has only been recently described in invertebrate species, and there have been only a few studies on its role in the reproductive system (Takacs-Vellai et al., 2005; Marsden et al., 2007; Barth et al., 2011; reviewed in Yin et al., 2017). In *Caenorhabditis elegans* and *Drosophila* species, starvation-induced autophagy was shown functionally involved in the development of germ cells and follicular cells during ovarian maturation (Barth et al., 2011). Recently, in our studies by autophagic genes mining and bioinformatics analyses in *M. rosenbergii*, the presence of major autophagy proteins has been identified (Suwansa-ard et al., 2016). Also, short-term starvation induced autophagy and stimulated ovarian maturation in female *M. rosenbergii* (Kankuan et al., 2017). The involvement of stress-induced autophagy in Sertoli cell’s survival and spermatogenesis has been studied in various species (Yin et al., 2017). However, the effects of starvation on autophagy in reproductive organs in male crustaceans are still unknown. Thus, the objective of this study was to investigate the effects of starvation on testicular maturation and spermatogenesis and the possible association with autophagy in the testes of male *M. rosenbergii.* We found that short-term starvation promoted testicular maturation and sperm production, and concurrently upregulated autophagy in testicular cells. The present findings contribute to better understanding the relationship between feeding-starvation and reproduction as well as the possible role of autophagy in reproduction, which could translate into a non-invasive treatment of the male broodstock to stimulate their reproduction for stimulating the aquaculture of this edible prawn.

## Materials and Methods

### Experimental Animals

Adult blue-claw male *M. rosenbergii*, weighing 102 ± 8.57 g were obtained from a local farm in Ayutthaya province, Thailand. The prawns were held in circular tanks and acclimatized for 1 week before the experiments. During this period, the unhealthy prawns could not survive, whereas inactive prawns were excluded from the experiment. Then, they were split into fed and starved groups, each with 24 prawns. They were kept in plastic tanks filled with fresh water at a photoperiod of 12:12 h light and dark. About 30% of the water volume was changed every 2 days.

### Experimental Design

The prawns were either fed with commercial food pellets (Sunshine, Bangkok, Thailand) or starved for 12 days. At days 1, 4, 8, and 12, six prawns were randomly selected from each group, anesthetized on ice water, and sacrificed. The testis was collected from each prawn, and one-half was prepared for histological examinations, while the other half was kept at −80°C for Western blot analyses of LC3 levels.

### Preparation of Testes for Histological Examinations

Small pieces of the testes were immediately immersed in cooled 4% paraformaldehyde fixative overnight. The specimens were then dehydrated in increasing concentrations of ethanol, cleared in xylene, and infiltrated with paraffin, using a Leica TP 1020 automated tissue processor. The paraffin-embedded tissue blocks were cut at 5 μm thickness using a Leica RM 2235 rotary microtome and placed onto silane-coated slides. For hematoxylin and eosin (H&E) staining, tissue sections were first deparaffinized in xylene and rehydrated in descending concentrations of ethanol. Then, they were stained with Mayer’s hematoxylin for 15 min, counterstained with Eosin for 1 min, and mounted in Permount (Bio-Optica, Milan, Italy). The sections were observed under a Nikon E600 light microscope, and images were recorded with a Nikon DXM 1200 digital camera using an ACT-1 program (Japan).

### Determination of Germ Cell Proliferation by Ki67 Staining

The testis slides were deparaffinized in xylene, rehydrated in decreasing concentrations of ethanol, and washed with 0.1 M PBS. Subsequently, the epitopes of Ki67 protein were opened by incubation with warm 0.01 M citric acid for 30 min, free aldehyde groups were blocked with 1% glycine in 0.1 M PBS for 30 min at room temperature, and nonspecific bindings were blocked with a 10% blocking serum for 2 h at 4°C. They were then incubated overnight at 4°C with rabbit anti-Ki67 (Abcam) at a dilution of 1:200 in 5% blocking serum, and then with goat anti-rabbit secondary antibodies (Invitrogen) at a dilution of 1:5,000 in 5% blocking serum for 2 h at room temperature. Finally, the color was developed with NBT/BCIP solution kit (Roche). The negative controls were performed by omitting the primary antibody.

### Ultrastructural Examinations of the Testes

Small pieces about 1 mm^3^ of the testes were fixed in 2.5% glutaraldehyde and 2% paraformaldehyde in 0.1 M PBS pH 7.4 at 4°C overnight and posted-fixed in 1% osmium tetroxide (OsO4) in 0.1 M PBS for 2 h at 4°C. Subsequently, they were washed with cool distilled water, dehydrated in increasing concentrations of ethyl alcohol, and infiltrated twice with propylene oxide for 30 min each. Samples were then infiltrated with a mixture of propylene oxide and Araldite 502 resin (Electron Microscopic Science, Hatfield, PA) at a ratio of 2:1 for 30 min, 1:1 for 30 min, and 1:2 overnight, embedded in pure Araldite 502 resin, and polymerized at 45 and 60°C, for 2 days each. In preparing semithin plastic sections, the specimen blocks were cut at 600 nm thickness using a Sorvall MT-2 ultramicrotome, stained with 1% methylene blue, and observed under a Nikon E600E light microscope, and images were recorded with a Nikon DXM 1200 digital camera. To prepare ultrathin sections for transmission electron microscopy (TEM), the specimen blocks were cut at 70 nm thickness, mounted on 200-mesh copper grids, counterstained with lead citrate and uranyl acetate, and observed under a FEI-TECNAI 20 TWIN transmission electron microscope operating at 75 kV.

### Analyses of the Expression Levels of LC3 by Western Blot

Fresh testicular tissues were homogenized and sonicated in a lysis buffer (0.2% C_24_H_39_NaO_4_, 1 mM Na_3_VO_4_, and 50 mM NaF). Homogenates were centrifuged at 10,000 *g*, and supernatants were collected for Western blot analyses, and protein concentrations were measured using Bradford reagent (Sigma-Aldrich). Subsequently, an aliquot with 30 μg of total proteins was separated on 15% SDS-PAGE gel and transferred to a PVDF membrane (Millipore Corporation). The membranes were incubated in a blocking buffer containing 5% skim milk for 2 h at room temperature and then they were incubated overnight at 4°C with the rabbit anti-microtubule-associated protein 1 light chain 3 (LC3) primary antibodies (Sigma-Aldrich) at a dilution of 1:500. Immunoreactive bands were detected using an enhanced chemiluminescent substrate (Thermo Scientific) and exposed to Amersham Hyperfilm ECL (GE Healthcare Life Sciences).

### Statistical Analyses

The data were expressed as a mean ± S.E., and the data of starved groups were then compared with those of the fed groups using a one-way ANOVA (MU-SPSS version 18) and a *post hoc* Duncan’s test to verify the differences. A probability value of less than 0.05 (*p* < 0.05) indicated a significant difference.

## Results

### Effects of Starvation on Histology of Testes, Diameter of Seminiferous Tubules, Testis-Somatic Index, and Maturation Stages of Seminiferous Tubules

**Figure 1:**
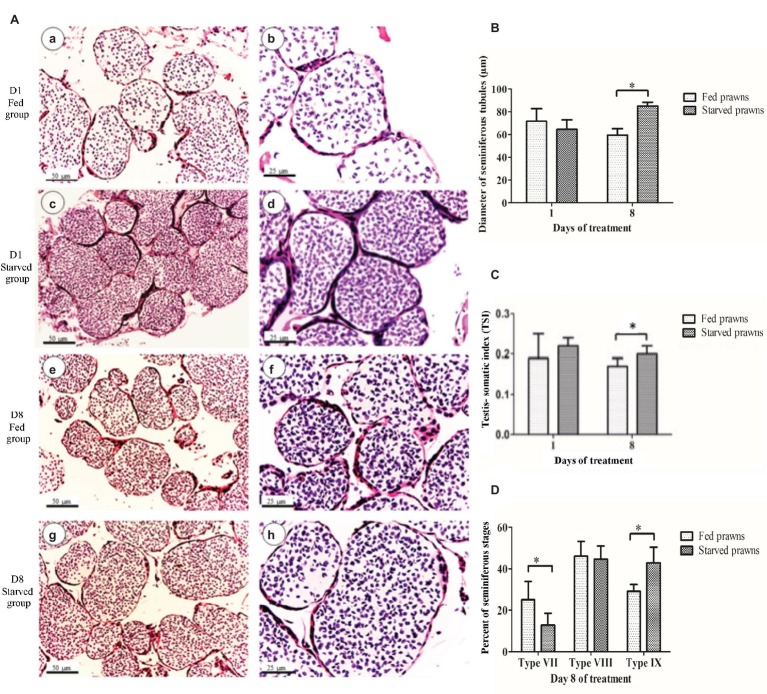
The effect of starvation on testes at days 1 and 8 as indicated by testicular histology **(A)**, testis-somatic index (GSI) **(B)**, mean diameters of seminiferous tubules (DST) **(C)** and maturation of seminiferous stages **(D)**. **(A)** Light micrographs showing seminiferous tubules in testes (H&E staining) at day 1 of fed prawns **(a** and **b)** and starved prawns **(c** and **d)**, and at day 8 of fed prawns **(e** and **f)** and starved prawns **(g** and **h)**. **(B)** Histograms showing testis-somatic index (TSI) of fed and starved prawns. **(C)** Diameters of seminiferous tubules (DST) in testes of starved and fed prawns at days 1 and 8. **(D)** At day 8, the testes of fed prawns showed more seminiferous tubules at middle stage VII than starved prawns, while the latter showed more seminiferous tubules at terminal stages IX. Asterisk indicates significant difference (*p* < 0.05) between the two groups (*n* = 5).

Microscopic examinations showed histology of the testes of fed and starved prawns at day 1 (Figures 1Aa–Ad, respectively) and day 8 (Figures 1Ae–Ah, respectively) comprised several lobes, and each lobe contained many seminiferous tubules (ST). The ST of the starved prawns appeared larger and contained more tightly packed cells than those of the fed prawns at both dates. However, when the diameters of their cross sections were measured, the difference of size was statistically different only at day 8 (Figure 1B). When we took a look at the testis-somatic index (TSI = TW/BW × 100), which is a measurement of the relative weight of the testis (TW) with respect to the body weight of the prawn (BW), we found that at both days 1 and 8, starved prawns had increased TSI compared to fed prawns and again the difference was statistically significant only at day 8 (Figure 1C). When the maturation stages of the ST were examined according to the criteria described by Poljaroen et al., 2010, at day 1, the percentages of seminiferous stages of starved group were not different from those of fed groups. On the other hand, at day 8, the testes of fed prawns showed mostly ST at middle stage VII, while the testes of starved prawns showed mostly ST at terminal stages IX which contained more spermatozoa than stage VII (Figures 1Ae,Af,D).

### Effects of Starvation on Germ Cell Proliferation

**Figure 2:**
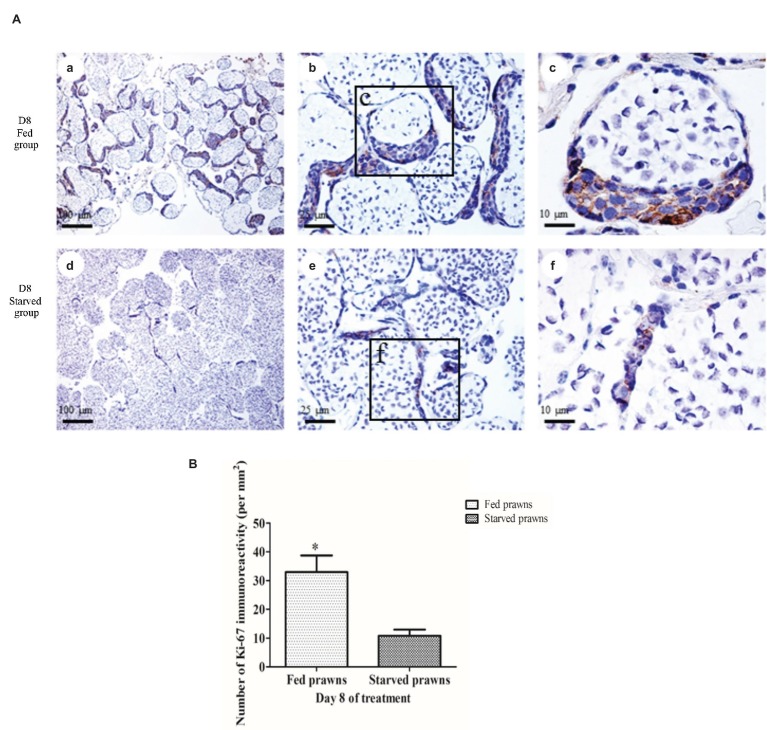
The effect of starvation on cell proliferation. **(A)** Light micrographs of the testes of fed and starved male prawns showing anti-Ki67 staining of dividing cells in the seminiferous tubules. The numbers of anti-Ki67-stained germ cells at day 8 were higher in the crescentic areas of the tubules of fed prawn (**a–c**) compared to those of starved prawns **(d–f)** whose tubules contain smaller crescentic areas with fewer number of anti-Ki67-stained germ cells. **(B)** Comparison of the numbers of Ki67-positive germ cells between fed and starved groups at day 8. Asterisk indicates significant difference (*p* < 0.05) between the two groups (*n* = 5).

The cell proliferation index, as monitored by immunostaining of Ki67 in the dividing germ cells, was estimated in the ST of both fed and starved prawns (Figure 2A). The Ki67-labeled cells were present in the crescentic area of each tubule which contained early germ cells (Poljaroen et al., 2010), while the remaining part of the tubule contained spermatids and spermatozoa which were not stained by anti-Ki67 (Figures 2Ab,Ac). The number of anti-Ki67-labeled cells at day 1 in these crescentic areas of the tubules was not different between fed and starved groups. On the other hand, when comparing the anti-Ki67-stained testes of fed prawn (Figures 2Aa–Ac) to that of starved prawns (Figures 2Ad–Af) at day 8, there were more anti-Ki67-stained germ cells in the crescentic areas of the former, while the latter contained smaller areas of crescentic areas with fewer anti-Ki67-stained germ cells. The comparison of the numbers of Ki67-positive germ cells at day 8 in the testes of fed and starved prawn was shown quantitatively in Figure 2B, and the difference was statistically significant.

### Microscopic Analyses Support a Functional Association Between Spermatogenesis and Autophagy Induced by Starvation in the Testis

**Figure 3:**
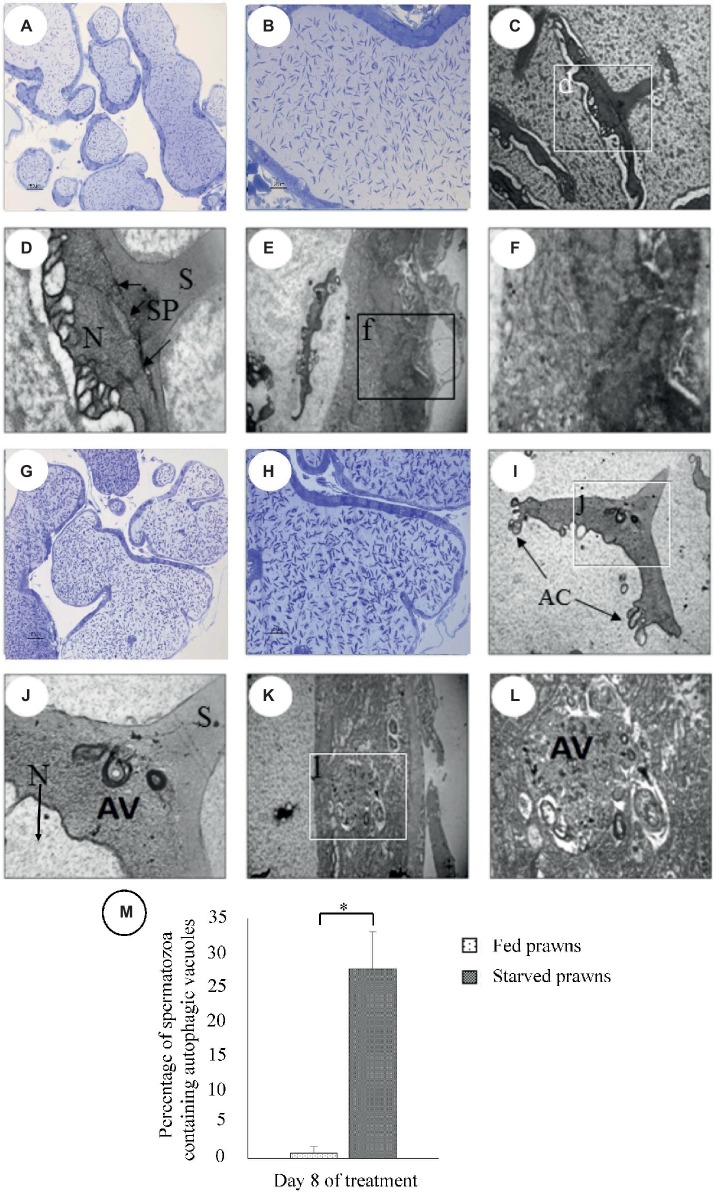
Micrographs of semi-thin and ultra-thin sections of testes of fed **(A–F)** and starved **(G–I)** prawns at day 8 as viewed by transmission electron microscope (TEM). Semithin sections of testes stained with methylene blue showed increased numbers of spermatozoa in seminiferous tubules of starved prawn **(G,H)** more than in fed prawns **(A,B)**. TEM micrographs of the testes of fed prawn show sperm **(C,D)** and cells forming the wall of seminiferous tubules **(E,F)** with normal ultrastructure. In contrast, the testes of starved prawn showed sperm **(I,J)**, cells in the wall of seminiferous tubules **(K,L)** containing autophagic vacuoles (AV), and percentage of spermatozoa containing AV **(M)**. N, nucleus; S, spike; SP, spike’s base plate; AC, acrosomal sacs. Asterisk indicates significant difference (*p* < 0.05) between the two groups (*n* = 5).

In searching for a possible involvement of autophagy in the starvation-induced effects on the seminiferous tubules observed in the testis of starved prawns, we performed a parallel microscopy analysis under light microscope (LM) and transmission electron microscope (TEM) to detect the number of spermatozoa in the ST and the presence of autophagic vacuoles inside the spermatozoa and testicular cells, respectively. To this end, semithin sections of testes from prawn starved at day 8 were prepared, stained with methylene, and observed by LM. The images in Figure 3 show an increased number of spermatozoa, which appear tightly packed in seminiferous tubules (Figures 3G,H), as compared to what was observed in the samples from the fed counterpart (Figures 3A,B). Notice also smaller crescentic areas that contained early germ cells in seminiferous tubules of starved prawns’ testes. Ultrathin sections of the testes of fed prawns as observed by TEM revealed the presence of spermatozoa (Figures 3C,D) and cells forming the wall of seminiferous tubules which were early germ cells (Figures 3E,F) with normal ultrastructure. In contrast, the testes of starved prawn presented spermatozoa (Figures 3I,J) and cells in the wall of seminiferous tubules (Figures 3K,L) that contained an increased number of autophagic vacuoles (AV). In the spermatozoa, the AV were present in the cytoplasmic area under the spike (Figures 3I,J, arrow), and the percentage of spermatozoa containing AV in starved prawns was significantly increased compared to fed prawns (Figure 3M).

### Effect of Starvation on LC3 Expression in the Testes

**Figure 4:**
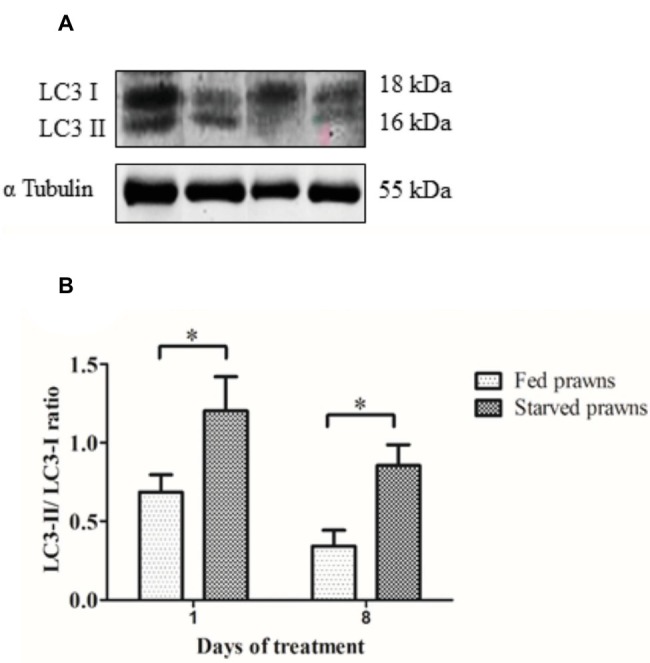
The effect of starvation on the expression of autophagy markers in the testes. **(A)** Immunoreactivity of testes from fed and starved prawns at days 1 and 8 showed bands of LC3-I and II as probed by anti- LC3. Immunoblots of α tubulin was used as a control. **(B)** The ratio of LC3-II to LC3-I intensity was increased in the testicular extracts from starved prawns (S) when compared to those of the fed (F) prawns at both days 1 and 8. Asterisks indicate significant difference (*p* < 0.05) between the two groups (*n* = 5).

The above data suggest that induction of autophagy parallels the increased spermatogenesis observed in the testes of starved prawns. To have a biochemical confirmation of the induction of autophagy, samples of control and starved testes were processed for Western blotting assessment of the autophagy marker LC3. During autophagosome formation, the MAP-LC3 precursor is first processed into soluble LC3-I and thereafter into the membrane-associated LC3-II peptide isoforms. The latter is considered a reliable hallmark of the presence of autophagic vacuoles (autophagosomes and autolysosomes) in the cell (Klionsky et al., 2016). Homogenates from testes of either starved or fed prawns showed the two LC3 immunoreactive bands, the upper band of molecular weight of 18 kDa corresponding to the cytosolic LC3-I isoform and the band at molecular weight of 16 kDa corresponding to the membrane-associated LC3-II isoform. It was clearly evident that in the starved testis, the amount of LC3-II relative to LC3-I was increased both at days 1 and 8, compared to the LC3-II/LC3-I ratio in the testes fed prawns (Figures 4A,B).

## Discussion

In this study, we report on the effect of nutrient starvation on the testes of the blue-claw male *M. rosenbergii*. We found that the testes of the prawns subjected to such metabolic stress present with early signs of testicular maturation, as signified by the increase of TSI, diameter of seminiferous tubules (DST) values, and number of germ cell proliferation. These signs resemble those seen when the animals are primed by gonadotrophic agents (Poljaroen et al., 2011).

Intriguingly, these changes were associated with the presence of autophagy vacuoles in the spermatozoa and early germ cells with upregulation of the expression of LC-3-II, a lipidated protein that marks the autophagy vacuoles.

A brief starvation could stimulate early gonadal maturation both in plants, as reported in tropical fruits such as mango (*Mangifera indica* L.) and mangosteen (*Garcinia mangostana* L.) (Poerwanto et al., 2008) as well as in animals such as in male rats (Bergendahl and Huhtaniemi, 1993) and in obese women (Van Dam et al., 2002). This is probably a compensatory mechanism for the survival of the species. In prawns, our recent study has shown that brief starvation in female *M. rosenbergii* could stimulate ovarian maturation and oogenesis (Kankuan et al., 2017). In this report, we found that male prawns reacted similarly to starvation as female prawns. A brief period of starvation, in this case not more than 8 days, could stimulate the increase of testicular weight with respect to body weight.

Similar to mammals, the testis of prawn is composed of seminiferous tubules which were classified into nine stages (stages I–IX) based on the proportion and associations of various steps of germ cells. Spermatogonia and nurse cells were present in all stages, while primary spermatocytes, spermatids, and mature sperm were of different proportions in each stage (Poljaroen et al., 2010, 2011). Stages I–V were classified by six phases of primary spermatocytes. Stage I contains mostly leptotene spermatocytes; stage II contained mostly zygotene and pachytene spermatocytes; stage III contained mostly diplotene spermatocytes; stage IV contained mostly diplotene and metaphase spermatocytes; and stage V contained mostly metaphase spermatocytes. Stages VI–VII contained mostly spermatids, while stage VI contained mostly early and mid-stage of spermatids, and stage VII contained mostly late stage spermatids. Stage VIII contained mostly premature sperm with condensed chromatin, and stage IX contained mostly mature sperm with decondensed chromatin. Therefore, stages VIII–X are considered the late or maturing stage with a consecutive increase of mature spermatozoa with the decrease of early germ cells in the crescentic zone of each tubule. In this report, we observed the early shift to the final maturation stages in the starved prawns compared to the fed prawns, with a consecutive decrease of early germ cells and their division (as reflected by the numbers of Ki-67 labeled cells) in the crescentic zone of the seminiferous tubules.

It is well known that starvation can induce the autophagy process resulting in the digestion and degradation of self-protein and damaged organelles by the lysosome. Microtubule-associated protein 1A/1B-light chain 3 (MAP-LC3) is the mammalian homolog of yeast atg8. On induction of autophagy, a cytosolic form of LC3 (LC3-I) is conjugated with phosphatidylethanolamine to form LC3-phosphatidylethanolamine conjugate (LC3-II), which is post-translationally inserted into the bilayer of the inner and outer membranes of the autophagosome (Kabeya et al., 2004). Autophagosomes fuse with lysosomes to form autolysosomes wherein the lysosomal hydrolases degrade intra-autophagosomal components (Kabeya et al., 2004; Klionsky et al., 2016). The rate of conversion of LC3-I into LC3-II can be assumed as a maker for monitoring autophagosome formation, and the presence of LC3-positive vacuoles is a reliable indicator of ongoing autophagy (Klionsky et al., 2016). Autophagy can be further assessed by detecting the presence of autophagy vacuoles by electron microscopy (Klionsky et al., 2016).

Recently, our group reported the presence of mRNAs of several autophagy genes in the transcriptome and the expression of the corresponding proteins in various tissues of the giant freshwater prawn *M. rosenbergii* (Suwansa-ard et al., 2016). This study showed that amino acid sequence and structural conformation of *M rosenbergii* LC3 were similar to that of human LC3, demonstrating that this autophagy protein is highly conserved not only in the vertebrates, but also in crustaceans (Suwansa-ard et al., 2016). More recently, we found that antibody against mammalian LC3 can cross-react with the two isoforms of the corresponding protein in prawns (Kankuan et al., 2017). Thus, the antibody of LC3 can be used as a marker for autophagic flux in crustaceans’ gonads. We have previously reported the hyper-expression of LC3-II protein and the presence of LC3-positive vacuoles in the ovary tissue associated with oocyte maturation in starved prawns (Kankuan et al., 2017).

In the present study, we show increased immunoreactivity of the autophagy marker LC3-II in the testis of starved prawns. Starvation-induced autophagy in the testis was further supported by ultrastructural study showing the occurrence of autophagy vacuoles in spermatozoa as well as in cells within the crescentic areas of the seminiferous tubules. This observation supports the hypothesis that autophagy induced by starvation is functionally involved in the remodeling that associates with testicular maturation and sperm production. It should be stressed that when starvation was prolonged to 12 days, the testis and germ cells underwent degeneration. It is conceivable that in chronic stressful condition, the sustained upregulation of autophagy led to excessive self-structure degradation with ensuing autophagic cell death. On the contrary, a limited (optimally 8 days) period of starvation seems to kick-start the testicular development, yet the refeeding may then be necessary to allow the continuation and bring to completion of testicular maturation and germ cell development. This hypothesis needed to be explored in the future as it has potential for exploitation in the increase of reproductive capacity of the male broodstock.

## Conclusions

We have found that a brief (up to 8 days) period of starvation in male *M. rosenbergii* has a stimulating effect on autophagy and spermatogenesis. The use of short-term starvation in stimulating male broodstock reproduction could possibly be applied in aquaculture where re-feeding after starvation may be employed to accelerate testicular maturation, leading to the enhancement of sperm quantity and quality, as well as fertilizability. As for the bio-reproductive relevance of the present findings, we propose the transient nutrient starvation stress as an effective non-cruel strategy to induce gonadal maturation in prawns in substitution of the current practice of eyestalk ablation and other invasive approaches.

## Data Availability Statement

All datasets generated for this study are included in the manuscript/supplementary files.

## Ethics Statement

This study was carried out in accordance with the recommendations of the Ethics Committee on the Use of Experimental Animals, Faculty of Science, Mahidol University.

## Author Contributions

WK performed the research and wrote the manuscript. ST and TS contributed to animal treatment and collection. FM and RT contributed to the Western blot analysis. MM and MN performed the transmission electron microscope. CW, PS, and CI provided conception, laboratory support, critical revision of the manuscript, and final approval of the version to be published.

### Conflict of Interest

The authors declare that the research was conducted in the absence of any commercial or financial relationships that could be construed as a potential conflict of interest.
